# Inhalation Exposure Analysis of Lung-Inhalable Particles in an Approximate Rat Central Airway

**DOI:** 10.3390/ijerph16142571

**Published:** 2019-07-18

**Authors:** Jingliang Dong, Jiawei Ma, Lin Tian, Kiao Inthavong, Jiyuan Tu

**Affiliations:** 1 Indoor Environment Engineering Research Center of Fujian Province, College of Ecological Environment and Urban Construction, Fujian University of Technology, Fuzhou, Fujian 350118, China; 2 School of Engineering, RMIT University, PO Box 71, Bundoora, VIC 3083, Australia

**Keywords:** CFPD, rat central airway, lung-inhalable particles, deposition enhancement factor, human, extrapolation

## Abstract

Rats have been widely used as surrogates for evaluating the adverse health effects of inhaled airborne particulate matter. This paper presents a computational fluid and particle dynamics (CFPD) study of particle transport and deposition in an approximate rat central airway model. The geometric model was constructed based on magnetic resonance (MR) imaging data sourced from previous study. Lung-inhalable particles covering a diameter range from 20 nm to 1.0 µm were passively released into the trachea, and the Lagrangian particle tracking approach was used to predict individual particle trajectories. Overall, regional and local deposition patterns in the central airway were analyzed in detail. A preliminary interspecies data comparison was made between present rat models and previously published human data. Results showed deposition “hot spots” were mainly concentrated at airway bifurcation apexes, and a gravitational effect should also be considered for inertia particles when using a rat as a laboratory animal. While for humans, this may not happen as the standing posture is completely different. Lastly, the preliminary interspecies data comparison confirms the deposition similarity in terms of deposition enhancement factors, which is a weighted deposition concentration parameter. This interspecies comparison confirms feasibility of extrapolating surrogate rat deposition data to humans using existing data extrapolation approach, which mostly relies on bulk anatomical differences as dose adjustment factors.

## 1. Introduction

A recent study concluded that over 87% of the world’s population lives in areas exceeding the safe annual aerosol exposure averaged level issued by the World Health Organization [1]. Adverse health effects of air pollution have long been recognized by epidemiological statistics, which indicate strong correlation between lung and heart disorders with particulate air pollution [2,3,4]. As a major component in air pollution, inhalable particulate matter (PM), particularly in the fine ranges (i.e., PM_2.5_ with diameter <2.5 μm), has been implicated as having a detrimental role in the pathogenesis of inflammatory lung disease, such as chronic obstructive pulmonary disease (COPD) [5,6].

Due to health risks involved in conducting experiments using human subjects, rats are widely used as surrogates for inhalation studies [7,8]. For exposure experiments, the three most commonly employed exposure routes are intra-tracheal instillation [9,10], nose-only exposure [11,12], and whole body exposure [13,14]. To obtain the registered toxicological response, most laboratory animal tests rely on excessively high exposure concentration levels [15,16]. While, environmental exposure levels are often much lower. A further challenge is determining precise regional exposure concentrations, which remains impossible due to the difficulty in performing accurate measurements for particle exposures with low deposition dose [17]. All these challenges pose great obstacles for toxicological analysis, which frequently involves dose-response assessment, interspecies data extrapolation, where accurate dose estimation is highly regarded [18].

Numerical simulations using computational fluid and particle dynamics (CFPD) can bypass these challenges and augment physiologic understanding when combining realistic respiratory conditions and anatomy. Compared to in vivo studies, computational models present an alternative solution to perform airway exposure studies in silico. This enables a cost-effective and more efficient way to predict regional deposition dose in the airway under various exposure conditions. Information such as detailed airflow patterns, spatial, and local distributions of deposited particles provides great assistance in establishing dose-response correlations for local tissue of concern.

Till now, numerous studies using respiratory airways of rat have been done, and most research efforts were spent on the upper respiratory airway (mainly the nasal passage). Kimbell and Godo [19] investigated gas uptake in a F344 rat nasal passage to determine the role of nasal airflow patterns that affect olfactory lesion distribution. The study provided the first description of flow in the complex olfactory region of a F344 rat. Minard and Einstein [20] presented airflow simulations by using proton magnetic resonance imaging of nasal-sinus passages in the rat. However, the anterior vestibule section of the nasal passage was omitted due to restricted imaging scope, and thus the particle filtration effect during inhalation was not fully included. Yang, Scherer [21] built a partial nasal cavity model (only included the right nasal cavity) based on nasal cast cross sections of a Sprague-Dawley rat. The study provided detailed velocity profiles, volumetric flow distributions, and streamline patterns.

Besides airflow dynamics analysis, local dose of inhaled gas or particles has also been extensively investigated. Kimbell and Subramaniam [22] numerically compared the regional inhaled formaldehyde uptake patterns in the nasal passage of multiple species, including F344 rat, rhesus monkey, and human. However, the rat and monkey nasal models were constructed from tissue specimens through tracing the outlines of the right nasal passages. Therefore, the anatomical quality of these models was largely compromised, especially in distal regions adjacent to ethmoid recesses. Later, Garcia, and Kimbell [17] adopted the same rat nasal passage model and conducted airflow and nanoparticle deposition simulations by using a Eulerian approach. The olfactory deposition revealed a peak olfactory dose with deposition efficiency of approximately 6% ~ 9% for 3- to 4-nm particles. More recently, Gu and Wen [23] numerically investigated the unsteady particle deposition characteristics during inhalation, and compared prediction results of inhaled micron particles with steady flow assumptions. In addition, previous studies by the authors [24,25] systematically investigated and compared the regional uptake of micron- and nanoparticles in the nasal cavities between a Sprague-Dawley rat and adult human, which offered valuable insights for inter-species data extrapolation.

Compared to the research attentions attracted by the upper respiratory airway, available studies focusing on the rat pulmonary airflow and associated exposure dose in this region are sparse. A prior species comparison study was conducted by Corley and Kabilan [26], which presented fruitful information of the airway ventilation in rat, monkey, and human respiratory tracts. Gas (acrolein) uptake patterns were determined and direct inter species comparisons were performed. Approximately 17 generations of the airway were constructed in the rat lung model, with the lower airway geometries based on lung cast data due to the dimensions of distal airway branches being well below the resolution achievable by magnetic resonance (MR) or micro computed tomography (micro-CT). To capture realistic airway anatomy, Oakes and Scadeng [27] performed MR imaging of four male Wistar rat lungs and measured the geometric features of the first four airway generations, including airway length, diameter, gravitational, bifurcation, and rotational angles. In their following study [28], particle transport and deposition patterns of 0.95 µm particles were numerically investigated for steady and unsteady cases.

Although numerous in-silico studies have been performed, research efforts were mainly spent on particle transport and deposition in rat nasal passages, where studies focusing on downstream central airways are scarce and existing exposure studies often neglect the pivotal upstream nasal filtration roles as demonstrated in our previous study [29]. To address this research challenge and advance our understandings about inhalation exposure characteristics in the rat central airway region, an approximate central airway model was reproduced [27] and CFPD simulations of lung-inhalable particles (ranging from 100 nm to 2.5 µm) under resting inhalation condition were conducted in this study. All particles were passively released into the trachea. The Lagrangian particle tracking approach was used to calculate individual particle trajectories. Overall, regional and local particle deposition patterns were analyzed in detail. More importantly, local deposition doses were compared with that in human lung airways.

## 2. Material and Methods

### 2.1. Rat Lung Airway Model

**Figure 1:**
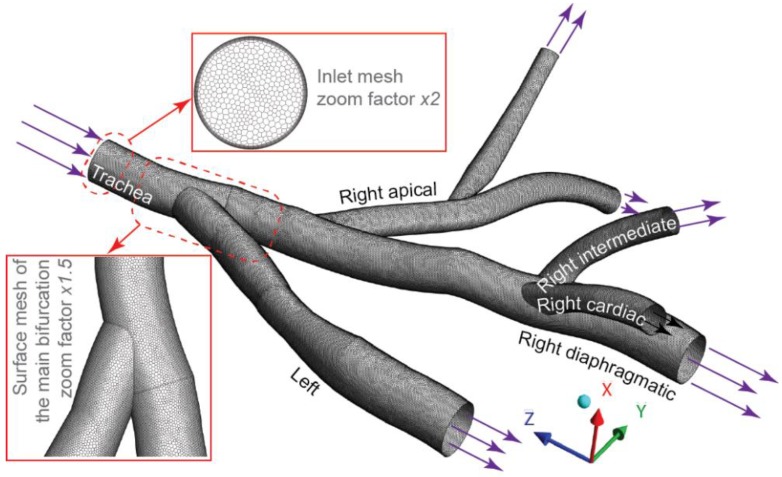
The rat lung airway model constructed from previous morphometric study [27].

Morphometric measurement data of four Wistar rat lungs reported by [27] was used to rebuild the present computational model. The data including information on path length, hydraulic diameter, bifurcation angle, and gravitational angle, which was based on MR images and averaged between four male rats. Based on this morphometric study, a 3D geometric central airway model for the first four airway generations of the rat lung was constructed. The procedure involved drawing centerline paths, creating lumen contours perpendicular to the path, and then lofting the lumen contours to create the final 3D geometric model (Figure 1). Each conducting airway was identified by its corresponding lobe respectively. The resulting central airway model consists of five main conduits connected to downstream lobes, including the left lung, right apical lobe, right diaphragmatic lobe, right intermediate lobe, and right cardiac lobe.

### 2.2. Numerical Modeling

The central airway model was meshed with polyhedral elements using ANSYS Fluent Meshing (ANSYS Inc., Lebanon, NH, USA). Prism layers were applied in near wall regions to capture accurate particle near wall behavior (Figure 1). The independent mesh results contained 1.6 million cells with approximately 8.0 million faces. Physiologically relevant flow conditions reported by Oakes, Marsden [28] were adopted in present study, where a constant mean inhalation flow rate of 0.44 L/min (equals to 7408 mm^3^/s) was set at the inflow trachea face as the inlet boundary condition, and outflow divisions among five lobes were set as: Apical—11.9%, Intermediate—18.1%, Diaphragmatic—27.9%, Cardiac—10.4%, and Left—36.4% according to subtending lobe volume percentage.

Lagrangian discrete phase model (DPM) including drag, gravity and Brownian forces was used to predict particles transport behavior within the nasal passage. For a dispersed phase (particles) with low volume fraction, the one-way coupled Lagrangian approach was used [30,31], where the airflow field was first simulated, and then the trajectories of individual particles were tracked by integrating the particle force balance equation,

(1) $$\frac{du_{i}^{p}}{dt}=f_{D}+f_{G}+f_{B}$$

The subscript and superscript *p* refers to the particle phase; $f_{G}$ is the gravity force (in negative *x*-direction); $f_{D}$ is the drag force per unit particle mass taking the form of Stokes’ drag law [32] defined as,

(2) $$f_{D}=\tau_{p}\left(u_{i}^{g}-u_{i}^{p}\right)$$

where $\tau_{p}$ is the particle response time, $\tau_{p}=18\mu /d_{p}^{2}\rho_{p}C_{c}$; and *C_c_* is the Cunningham correction factor to Stokes’ drag law, which is calculated from,

(3) $$C_{c}=1+\frac{2\lambda }{d_{p}}\left(1.257+0.4e^{-\left(1.1d_{p}/2\lambda \right)}\right)$$

here, *λ* is the mean free path of air molecular. The components of the Brownian force were modelled as a Gaussian white noise process with spectral intensity [33]

(4) $$S_{n,ij}=S_{0}\delta_{ij}$$

where *δ_ij_* is the Kronecker delta function, and *S_o_* is a spectral intensity function,

(5) $$S_{0}=\frac{216\nu k_{B}T}{\pi^{2}\rho d_{p}^{5}{\left(\frac{\rho_{p}}{\rho }\right)}^{2}C_{c}}$$

In present study, particle density *ρ_p_* was set as 1000 kg/m^3^, and air density *ρ* was set as 1.225 kg/m^3^. *T* is the absolute temperature of the inhaled air (in present study 293 K), *ν* is the kinematic viscosity, and *k_B_* is the Boltzmann constant. Amplitudes of the Brownian force components are of the form

(6) $$f_{B}=\zeta \sqrt{\frac{\pi S_{0}}{\Delta t}}$$

where *ζ* is a zero mean, unit variance independent Gaussian random numbers, and ∆*t* is the particle integration time-step.

Our previous study [29] revealed 20 nm to 1.2 µm particles could pass through the nasal passage smoothly without major deposition loss (deposition efficiency less than 10%). This indicates that most particles in this size range can be conveyed into the lower respiratory airways. Thus, in the present study, selected particle sizes with a diameter value of 20, 40, 70, 100, 200, 500, 800, 950, and 1000 nm were used for central lung airway exposure studies, and inhalation flow rate was set as 0.44 L/min. For each particle size, 100,000 particles were passively released at the inlet of trachea to ensure the deposition results are independent of particle numbers. The airflow was considered incompressible with constant air density and all airway walls were treated as no-slip boundary condition. Since the peak Reynolds number was less than 200 (reference value extracted from fully developed flow at trachea section), the airflow was treated as laminar. All numerical simulations were conducted by using ANSYS-Fluent v17.0 (ANSYS Inc., Lebanon, NH, USA), and all residual values less than 10^−4^ was set as the simulation convergence criteria.

## 3. Results and Discussion

### 3.1. Airflow Patterns

**Figure 2:**
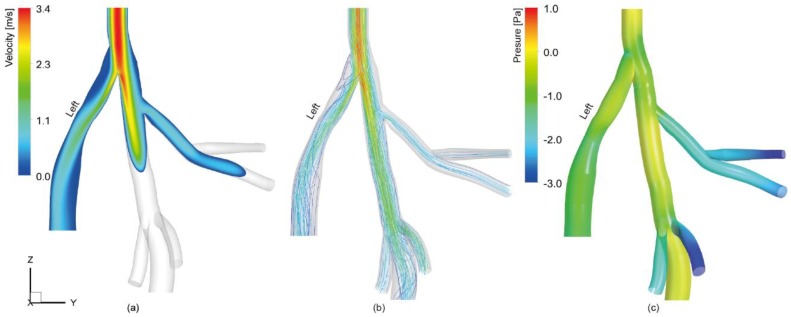
Airflow patterns in the rat lung airway: (**a**) Velocity magnitude contour in the center plane of the airway model; (**b**) airflow streamlines; and (**c**) surface pressure showing the pressure drop along the airway. Note, due to the irregular shape of the airway anatomy, velocity distributions in the lower half of the central airway were not presented in Figure 2a, but this can be viewed from velocity-colored streamlines in Figure 2b.

Airflow patterns including velocity contours at the center plane, streamlines, and surface pressure were presented in Figure 2. Figure 2a shows a parabolic (fully developed) flow profile at the trachea entrance, with a maximum core velocity of 3.4 m/s. The flow bifurcated into two streams at the carina of trachea (the tracheal bifurcation site). Due to the asymmetric volume between the left and right lung (volume ratio of 3:5 based on the adopted lobar volume values), more airflow was diverted into the right lung with the bulk flow concentrated along the inner wall of the bifurcation (core velocity 2.5 m/s). For the left lobe airway, disturbed separated flow and its reattachment was observed along the outer airway wall, and the core velocity gradually decreased from 2.5 m/s to less than 1.0 m/s as approaching to its distal end. Airflow in the right apical lobe exhibited a more developed velocity contour with a center velocity of 1.0 m/s in the 1st generation airway. Figure 2b depicts the flow streamlines, where notable flow disturbance was observed in the left lobe airway close to the main bifurcation and the right diaphragmatic lobe airway close to the trifurcation in the right lung. Apart from these disturbed flow regions, the inhaled air generally flowed smoothly in the airway segments. Lastly, Figure 2c shows the static pressure distribution along the airway. Peaking pressure was found at the carina of trachea as it acts as a flow divider during inhalation. It is also worth noting that the pressure distribution along the main bronchioles of the right lung is stable compared to that in other airway branches. This is mainly attributed to the relatively unchanged cross-sectional area and volume flow rate along the airway path. However, due to airway size reduction, notable pressure drops were observed at the distal branches of the right apical airway and the right intermediate airway.

### 3.2. Particle Deposition Patterns

**Figure 3:**
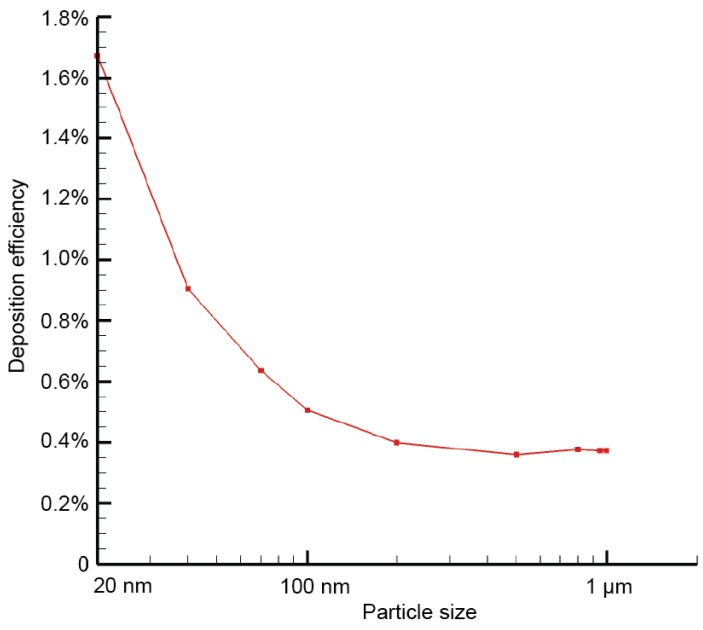
Overall deposition efficiency profile of all considered fine particle sizes.

Overall deposition efficiencies (the number of deposited particles over the total number of inhaled particles) in the present central airway model were summarized in Figure 3. In general, predicted deposition efficiencies for all considered particles ranging between 20 nm and 1 µm were extremely low (less than 1.8%). This indicates most selected lung-inhalable particles can be smoothly conveyed through the present central airway model without major deposition loss. The deposition efficiency profile peaks at 1.7% for 20 nm particles and then rapidly drops down to 0.5% for 100 nm particles. For particles larger than 200 nm, their deposition efficiencies remained relatively unchanged at 0.4%.

**Figure 4:**
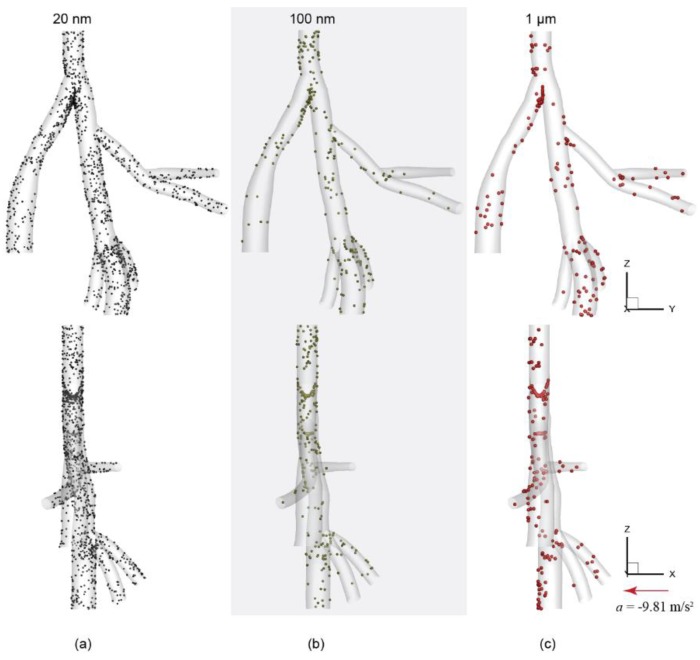
Particle deposition patterns comparison with particle sizes of (**a**) 20 nm, (**b**) 100 nm, and (**c**) 1 µm. Note, for better illustration purpose, particle symbol size was slightly increased for larger particles.

Spatial deposition patterns were presented in Figure 4 based on selected particle groups with size of 20 nm, 100 nm, and 1 µm. Results showed that deposited particles tended to concentrate around the bifurcation apex (carina), and along the posterior wall in distal right diaphragmatic airway (Figure 4c). For 20 nm particles, deposited particles were scattered over the whole 3D geometry, with obvious particle pileups at the carina of trachea and the trifurcation region near the distal right diaphragmatic airway. This was consistent with the observed disturbed flow regions in Figure 2b. For 100 nm particles, deposited particles were significantly reduced due to reduced particle diffusion effects, which is inversely proportional to the particle diameter. For 1 µm, overall deposition patterns remain relatively unchanged, but more particles were deposited at the posterior side of the airway due to gravitational deposition.

**Figure 5:**
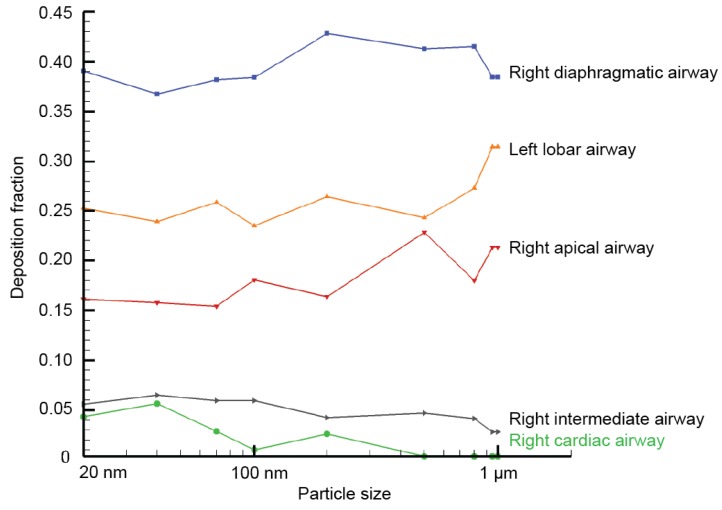
Compartment deposition fraction of the overall deposition in the central airway.

In Figure 5, the fraction of regional deposited particles over total deposited particles in the whole airway model was calculated for all five main airway compartments. Results show most particles were deposited in the right diaphragmatic branch, with a fraction of 0.36–0.42. This is followed by the fraction in the left lobar airway, where relatively stable fraction was seen for particles between 20 nm to 500 nm. Then, as particle size approaching to 1 µm, a sharp increase was observed, and the fraction value peaked at 0.31. The right apical airway exhibited a similar trend with relatively constant deposition fraction (0.16) for 20 nm to 200 nm. However, for particles larger than 200 nm, the regional fraction profile started to fluctuate, and peaked at 0.23 for particle size of 500 nm.

For right intermediate branch and right cardiac branch, the captured deposition fractions were all at minimal levels. The deposition fraction in the right intermediate airway decreased from 0.06 to less than 0.03 between 20 nm to 1 µm. Lastly, the right cardiac airway produced the lowest deposition fraction among all major branches, particular for particles greater than 500 nm, where particles were barely deposited.

**Figure 6:**
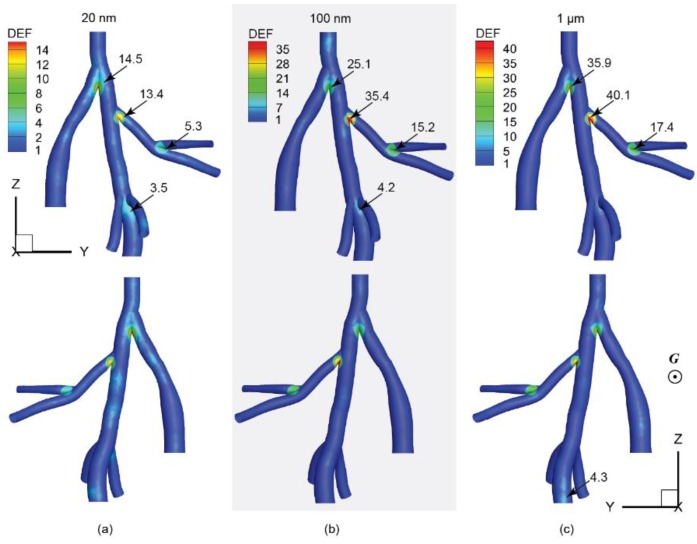
Deposition enhancement factors (DEFs) for the rat lung airway with particle sizes of (**a**) 20 nm, (**b**) 100 nm, and (**c**) 1 µm.

Many studies presented deposition enhancement factors (DEFs) to quantify the local deposition density within a defined area relative to overall deposition density in the entire region [34,35,36,37]. In this study, the local DEF was defined as:

(7) $$\mathrm{DEF}\;=\;\frac{\mathrm{number}\;\mathrm{of}\;\mathrm{particles}\;\mathrm{deposited}\;\mathrm{within}\;a\;\mathrm{fixed}\;\mathrm{radius}\;\mathrm{of}\;a\;\mathrm{surface}\;\mathrm{node}/A_{\mathrm{Local}}}{\mathrm{overall}\;\mathrm{number}\;\mathrm{of}\;\mathrm{particles}\;\mathrm{deposited}\;\mathrm{in}\;\mathrm{the}\;\mathrm{whole}\;\mathrm{airway}/A_{\mathrm{Total}}\;},$$

where $A_{\mathrm{Local}}$ is the local region with a fixed searching radius of 1 mm, $A_{\mathrm{Total}}$ is the overall surface area of all considered airway regions. DEF contours of typical particle sizes, 20 nm, 100 nm, and 1 µm, were shown in Figure 6.

In general, intensively packed particle deposition tends to concentrate around bifurcation apexes. For 20 nm particles (Figure 6a), the main bifurcation apex showed a peak DEF of 14.5, which is followed by the bifurcation apex between the right diaphragmatic and right apical airways, with a DEF value of 13.4. For particle size of 100 nm (Figure 6b), DEF values at bifurcation apexes were considerably increased compared to that in the 20 nm case. The maximum DEF of 35.4 was found at the bifurcation apex of the right diaphragmatic and right apical airway, which is nearly three-fold of that observed at the same location in the 20 nm case. While the second-most intensively packed region was found at the primary bifurcation apex with a DEF of 25.1. As for 1 µm particles (Figure 6c), the DEF distribution patterns were close to the 100 nm case. Where the peak DEF value of 40.1 was found at the bifurcation apex joining the right apical and right diaphragmatic branches, following by a DEF of 35.9 at the primary bifurcation apex. Note, DEF is a weighted value that aims to quantify the distribution inhomogeneity of deposited particles only. For particles with larger sizes, although a significant deposition magnitude loss was observed (Figure 3), the distribution unevenness of deposited particles also became worse (Figure 4). Therefore, higher DEF values were seen in airways exposed to larger sized particles.

## 4. Discussion

It is widely accepted that inhalable particulate matter (PM) plays a detrimental role in the pathogenesis of chronic obstructive pulmonary disease (COPD) as indicated by many research studies in this field [38,39]. While the impact of ambient PM on the health of exposed individuals, such as the development of pathological and functional changes in the lungs, remains unclear. To continue exploring the dose-response mechanism of inhaled toxins, surrogate rats are widely used as a laboratory animal model for pulmonary exposure studies. However, existing exposure protocols may not be able to achieve penetrating lung exposure, especially for those acute exposure studies designated for a short exposure period. One pivotal factor is the particle filtration effect exerted by the rat nasal passage. This has been demonstrated by the authors’ pervious work [29], which provided a thorough understanding of particle transport and deposition patterns in a rat nasal passage using the same inhalation conditions.

**Figure 7:**
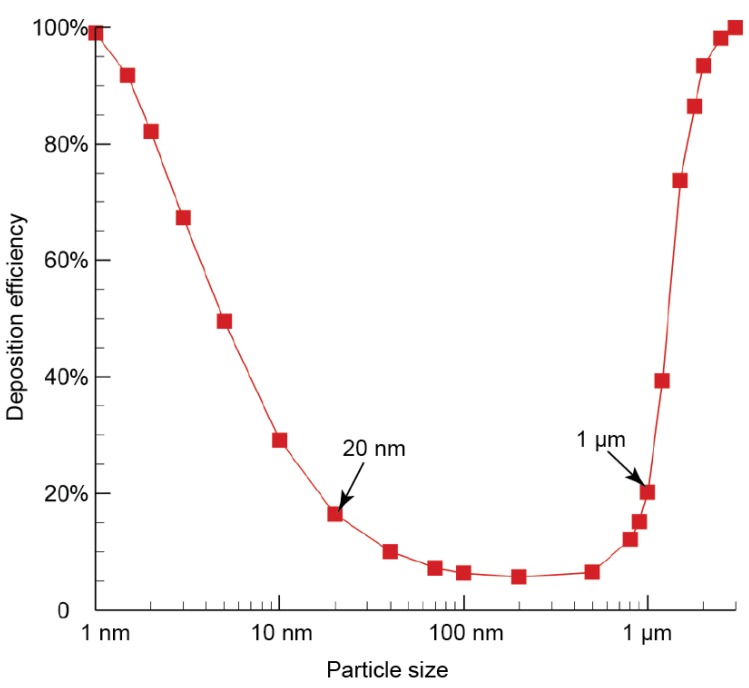
The overall particle deposition efficiency profile in a rat nasal passage. This deposition curve was reproduced from the authors’ previous work [29].

Based on the rat nasal deposition efficiency results (Figure 7) obtained from our previous study [29], the deposition profile follows a U-shaped trend. High deposition efficiencies tend to occur for either small diffusive particles (<20 nm) or large inertia particles (>1 µm) for all considered particle sizes ranging from 1 nm to 3µm. While fairly low deposition was found for particles ranging between 20 nm to 1 µm, indicating that inhaled particles in this size range can mostly pass through the nasal passage without major deposition loss. According to this research finding, particles ranging between 20 nm and 1 µm were selected in present work as representatives of lung-inhalable particulates.

**Figure 8:**
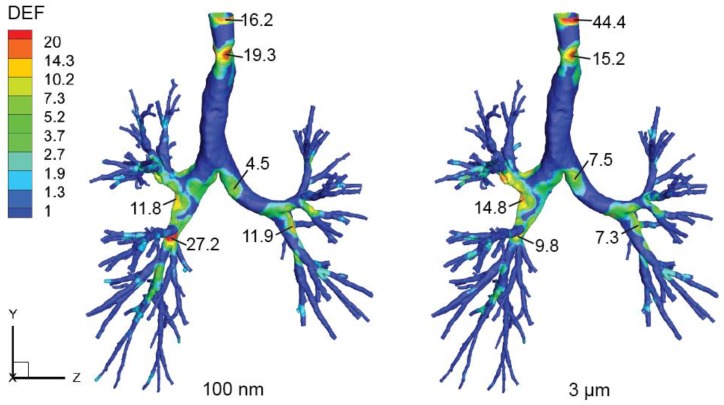
DEF contours of a human lower respiratory airway following exposure of 100 nm and 3 µm particles at flow rate of 18 L/min. Results were reproduced from [40].

To reveal the relevance of the present rat central lung airway model to human airways, a preliminary comparison was made between the DEF distribution in the present rat model and a human lung airway model reported in our recent work [40]. In that study, an extended human respiratory airway model was built, including both upper and lower respiratory airway compartments, and the human airway model was exposed to accumulation mode particles in the size range of 100 nm to 3.0 µm. In Figure 8, DEF contours of selective 100 nm and 3 µm particles under moderate inhalation flow rate of 18 L/min were presented for this preliminary comparison purpose. Although inertial deposition of 3 µm was adopted here, its spatial deposition was expected to be similar with 1 µm as no apparent change can be seen either from the overall deposition efficiency profile, or from the spatial deposition figures. Detailed deposition characteristics please refer to that paper.

As shown in Figure 8, the DEF distribution patterns in the human lower respiratory airway model were relatively similar between 100 nm and 3 µm particles, where highly packed particle depositions were found at narrowed site of the trachea airway lumen and bifurcation apexes, with biased particle deposition along the right primary bronchus. As for the comparison between rat and human models, in the light of keeping an anatomical scope similarity, the trachea deposition was excluded for interspecies comparison as no apparent trachea section was included in the rat model. In general, concentrated deposition (with relatively high DEFs) in the human model tends to occur at the 3rd and 2nd bifurcation apexes, and a peak DEF of 27.2 (100 nm case) was found close to the 3rd bifurcation apex of the right lung. While for the present rat central airway model, highly packed depositions were found at the 1st, 2nd, and 3rd bifurcation apexes, and the maximum DEFs were around 35 to 40. This preliminary interspecies comparison confirms that the bifurcation apex regions are prone to receive remarkable depositions in both rat and human lung airways. Despite the actual deposition density (or local dose) may differ vastly between these two species, the weighted deposition enhancement factor results showed comparable values with same order of magnitude for both species. More importantly, this resemblance further corroborates the existing data extrapolation approach, which mainly takes account of the bulk anatomical differences as dose adjustment factors [41].

## 5. Conclusions

This paper presented an in-depth analysis of particle transport and deposition patterns in a rat approximate central airway model by using selected lung-inhalable particles with a size range of 20 nm to 1 µm. The results showed the selected particle size can easily be transported through the airway model without major deposition loss. By introducing the deposition enhancement factor, both the deposition “hot spots” and its intensity compared to the overall averaged level can be effectively accessed. For deposited particles, major deposition “hot spots” were found at the vicinity of bifurcation apexes. As particle size increases, the overall deposition efficiency drops significantly. In the light of calculated DEF values, the largest DEF was found at the 2nd bifurcation apex (the joining point of the right diaphragmatic and right apical branches) at 40.1 for 1 µm particles, indicating a highly packed deposition at this region, with a density of 40 folds greater than the overall averaged level. The results also revealed a gravitational effect should also be considered for inertia particles when using a rat as a laboratory animal, as its posterior side of the airway received more particle deposition compared to the anterior side. While for human, this may not happen as the standing posture is completely different.

When estimating the potential risk of inhaled toxicants, there is an increasing emphasis on predicting the dose to the target tissue rather than total deposition rate. The findings of this study highlight the advantage of numerical modeling in predicting regional deposition patterns in the respiratory airway, and offers cost-effective estimation of local delivered dose when conventional measurements often could not resolve due to resolution restrictions. Further, the preliminary interspecies data comparison confirms the deposition similarity in terms of deposition enhancement factors, which is a weighted deposition concentration parameter. This interspecies comparison corroborates the applicability of some anatomical-based adjustment factors frequently used in present data extrapolation schemes.

Due to lack of relevant experimental inhalation exposure studies using rat lung airways, the numerical model presented in this paper was not validated against any measurement data. However, the numerical modeling strategy and most of the model configurations have been extensively validated in the authors’ previous studies, including rat nasal cavity [25], human nasal cavity [42,43], and human lung airways [36]. Those previous studies provided substantial benefits to the present work, which ensure consistent and rigorous numerical models.

Other limitations of this study include steady flow rates, rigid walls, and limited scope of the rat central airway. Future studies including more realistic physiological conditions and extended respiratory airway are necessary to continuously corroborate these findings. Despite these limitations, this study provides a detailed rat central airway exposure assessment to ambient fine particles, which may contribute important roles in preparing and conducting exposure experiments using laboratory rodents.
